# Improved Adhesion and Biocompatibility of Chitosan-Coated Super-Hydrophilic PVC Polymer Substrates for Urothelial Catheters

**DOI:** 10.3390/ijms26052128

**Published:** 2025-02-27

**Authors:** Alenka Vesel, Helena Motaln, Miran Mozetič, Dane Lojen, Nina Recek

**Affiliations:** 1 Jozef Stefan Institute, Department of Surface Engineering, Jamova cesta 39, 1000 Ljubljana, Slovenia; miran.mozetic@ijs.si (M.M.); dane.lojen@ijs.si (D.L.); nina.recek@ijs.si (N.R.); 2 Jozef Stefan Institute, Department of Biotechnology, Jamova cesta 39, 1000 Ljubljana, Slovenia; helena.motaln@ijs.si

**Keywords:** medical-grade PVC, chitosan coating, urinary catheter, urothelial cells, cytotoxicity

## Abstract

Chitosan is a water-soluble polysaccharide with good adherence to negatively charged surfaces and reported antimicrobial and anti-inflammatory properties. Coating the surfaces of medical devices with chitosan is a promising strategy for harnessing these benefits. However, the surface properties of commercial polymers need to be altered to enable the bonding of thin chitosan films. In this study, the adhesion of chitosan onto plasma-treated polyvinyl chloride (PVC) and the metabolic activity of urothelial cells on chitosan-coated medical-grade PVC used for the synthesis of urinary catheters were evaluated. To improve the adhesion of chitosan onto the PVC catheters, PVC samples were made “super-hydrophilic”. PVC substrates were briefly treated with a powerful hydrogen plasma and weakly ionised oxygen plasma afterglow to obtain a chlorine-free surface film, which was rich in oxygen functional groups, followed by incubation of the plasma-treated substrates in an aqueous solution of chitosan. Then, urothelial RT4 cells were seeded on the treated and untreated PVC substrates, and their metabolic activity, confluency, and cell morphology were examined. X-ray photoelectron spectroscopy was used to measure the nitrogen concentration, which corresponded to the chitosan concentration on the substrate. The results showed that the substrates were uniformly covered by a thin layer of chitosan only on plasma-treated surfaces and not on untreated surfaces. Moreover, the chitosan coating provided a stimulated environment for cell adhesion and growth. In conclusion, the chitosan-coated super-hydrophilic PVC substrate shows potential to improve the overall performance and safety of medical devices such as urinary catheters.

## 1. Introduction

Polyvinyl chloride (PVC) is a flexible polymer that is frequently used in various medical devices such as urinary catheters [1], infusion and artificial feeding devices [2,3], endotracheal tubes, and intravenous catheters [4]. However, these medical devices sometimes act as sources of hospital-acquired infections in patients [5,6,7]. The mortality rate of hospital-acquired infections is approximately 90,000 deaths per year [8], and these mortalities are associated with enormous costs [9]. Hospital-acquired infections include bloodstream infections (BSI) [10], catheter-associated urinary tract infections [11], and surgical site infections [12]. Although antibiotics are usually administered to patients to prevent such infections, antibiotic overuse has led to the emergence of superbacteria that are resistant to most antibiotics and other medications [13]. Therefore, the development of suitable antibacterial surfaces or antibacterial coatings is warranted as a strategy to mitigate this issue. Furthermore, the coatings should be as thin as possible and adhere well to the medical implants. Natural antimicrobial agents, such as chitosan, are good candidates for this application [14,15,16]. Chitosan is a polysaccharide that is soluble in water. Its solubility depends on its molecular weight and acetylation degree, as well as on the pH of the media. It contains amino groups in its structure, which enable its adherence to negatively charged surfaces. Numerous studies have reported the beneficial properties of chitosan [17,18,19,20,21,22]. For example, chitosan coatings have been used to coat endovascular catheters [23], dental implants [24,25], orthopaedic implants [21], titanium alloy implants [26] and stainless steel screws used for bone fixation [27], barrier membranes [28], wound dressing [29], and contact lenses [30,31].

Thus, coating the surfaces of medical devices with chitosan is a promising strategy for harnessing antimicrobial and anti-inflammatory properties, suppressing the frequency of hospital-acquired infections, and enabling better cell growth. The antibacterial activity of chitosan is a consequence of electrostatic interactions. It was reported that the positive charge of protonated amino group of chitosan interacts with the negatively charged molecules on the surface of bacteria [32]. This causes permeabilization of the cell surface and the leakage of intracellular substances. Its anti-inflammatory properties, on the other hand, arise from its ability to suppress inflammatory cytokines, increase anti-inflammatory cytokines, and regulate the level of oxidative stress [33].

Chitosan can also be used for coating urinary catheters. Urinary catheters are long flexible tubes that are often fabricated from medical-grade PVC polymers. Urinary catheters are used to empty the bladder of patients during surgery or to manage urinary incontinence [34]. Therefore, the firm attachment of any functional coating to the surface of the catheter is imperative to avoid its detachment from the surface, particularly during the insertion of the catheter into the body. Furthermore, non-satisfactory coating adhesion causes a risk of residuals from the coating remaining in the urethra after catheter removal, which may complicate the clinical application of coated PVC urinary catheters.

However, PVC is chemically inert and does not interact with chitosan; therefore, its surface properties need to be altered to enable the bonding of thin chitosan films. Several methods were reported for altering the surface properties of polymers to improve chitosan adhesion such as wet chemical treatments, ozone treatment, ion beam treatments, corona, and plasma treatments [35,36,37,38]. For example, Kurose et al. [39] exposed PVC to ozone to obtain a hydrophilic surface finish. Ozone molecules interact with PVC to form hydroxylic acids, ketones, and carboxylic groups. Gabriel et al. [40] used wet chemical treatment with ethylene diamine and observed a similar effect. Zhao et al. [41] dissolved atomic oxygen radical anions in distilled water and reported improved hydrophilicity, which could be attributed to the formation of oxygen-containing functional groups and dichlorination. Moreover, PVC hydrophilisation occurs on treatment with various organic chemical modifiers [42]. Ru et al. [43] performed Ar plasma treatment of PVC but only to deactivate radicals which remained after plasma polymerization of allylamine. Argon and oxygen plasma was also used by Ghoranneviss et al. [44]. The lowest contact angle that was obtained was 27°, which was explained by surface texturing. Similar contact angles were also obtained by Xiao-Jing et al. [45] who used nitrogen plasma. However, super-hydrophilicity was not reported. In the current study, we have used gaseous plasma treatment because it enables rapid functionalization with polar functional groups, which modulates the wettability of the super-hydrophilic surface finish. Opposite to the other authors mentioned above, we managed to obtain a super-hydrophilic surface that enabled chitosan adhesion.

The coatings based on various biocompatible materials are needed for modern biomaterials science [46] because of their promising applications in regenerative medicine [47], biosensors [48], advanced biomaterials [49], etc. Treatment of biological materials with non-equilibrium gaseous plasma is among the most promising techniques for tailoring the surface properties of materials used in modern medicine [50] because it enables modification of the surface functional groups without influencing the functional properties of treated materials [51]. Plasma treatment causes the formation of various surface functional groups but the methods for the formation of a specific group have yet to be discovered. The novelty of our approach was obtaining the super-hydrophilic surface finish without significant modification of the surface roughness. The super-hydrophilicity of polymers is usually attributed to a combination of polar surface functional groups and a rich morphology on the sub-micrometer scale [52]. In our case, the treated polymer retained a very smooth surface but still allowed chitosan to attach to the surface. A smooth surface is important to ensure good sliding properties and easier insertion of the catheter into the body.

The benefit of plasma treatment is in its ability to functionalize the surface with negatively charged groups to enable interactions with chitosan [53]. NH_2_ groups of chitosan can undergo protonation in acidic environments to form NH_3_^+^ groups [54]. These positively charged amine groups can electrostatically bind to negatively charged groups formed during plasma treatment [55]. Furthermore, plasma treatment also improves the wettability. Uniform surface wetting is another requirement for the formation of a uniform surface film on a polymer substrate. Oxygen plasma is most often used because it enables the formation of various groups including hydroxyl and carboxyl groups. Particularly, carboxyl groups are required for electrostatic interactions between the plasma-treated surface and chitosan. However, the effectiveness of the surface modification depends on the plasma treatment conditions and type of polymer used. Some polymers are unlikely to be functionalized with polar groups using standard treatment procedures, which may cause issues in inducing optimal adhesion properties. These polymers include halogen-containing polymers such as PVC.

**Figure 1:**
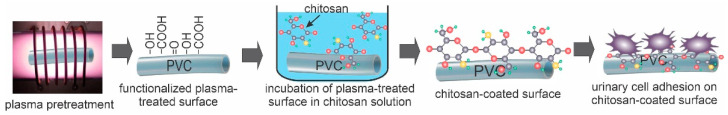
Overall concept of investigation.

In this study, we investigated two issues. One is applying gaseous plasma for surface functionalization of medical-grade PVC materials used in urinary catheters to achieve better chitosan adhesion. The second is examining the possible cytotoxicity of chitosan to prove its safe use. Two types of plasma treatments were used. One is a conventional treatment with oxygen plasma [56], whereas the other procedure involves a combination of pre-treatment with dense hydrogen plasma followed by treatment with mild oxygen plasma. We have recently shown that this combination is optimal for achieving a super-hydrophilic surface on polytetrafluoroethylene, which is a chemically inert material [57]. Then, we coated the plasma-treated PVC with an extremely thin film of chitosan and investigated the effect of chitosan on the viability of urothelial cells to validate its non-toxicity. The schematic concept of investigation of this study is presented in Figure 1.

## 2. Results and Discussion

### 2.1. Plasma Treatment of PVC Substrates

**Figure 2:**
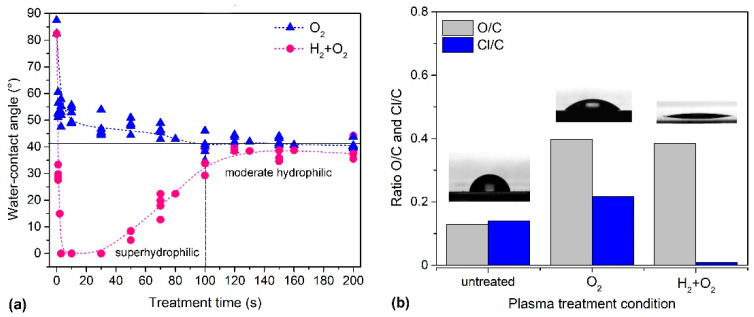
(**a**) Variation of water contact angle versus treatment time in oxygen plasma afterglow without pre-treatment (blue curve) and with H_2_ pre-treatment (red curve). (**b**) X-ray photoelectron spectroscopy (XPS)-based surface composition of selected plasma-treated samples (untreated, treated in oxygen plasma afterglow for 100 s (marked as O_2_), and pre-treated in hydrogen plasma for 1 s and treated in oxygen plasma afterglow for 3 s (marked as H_2_ + O_2_)) along with images of the respective water droplets.

In the first experiment, PVC samples were subjected to the standard treatment for hydrophilisation by exposure to an oxygen plasma flow afterglow. This treatment gradually reduced the WCA as treatment time increased. The WCA quickly reduced from 82° to <50° and stabilised to a minimum at approximately 40° (Figure 2a) after approximately 100 s of plasma treatment. Hence, 100 s was set as the treatment time for further PVC sample preparation steps before incubation with chitosan. Thus, the standard treatment with oxygen plasma afterglow only resulted in a moderately hydrophilic PVC surface. Therefore, a different method was sought to achieve better hydrophilicity.

In the second experiment, a pre-treatment step was performed using H_2_ plasma for sample activation. We have shown in a previous study that this treatment enables the fabrication of super-hydrophilic surfaces [58]. The duration of this pre-treatment step was only 1 s. H_2_ plasma is an intensive source of VUV (vacuum ultraviolet) radiation [59,60], which causes bond scission in the surface film, leading to the formation of reactive dangling bonds on the surface [61,62]. These dangling bonds are highly reactive towards hydrogen atoms. Therefore, hydrogen plasma pre-treatment elicits the formation of a very thin polyolefin-like film on the PVC surface.

Samples pre-treated with hydrogen plasma were immediately exposed to further treatment with O_2_ plasma afterglow for 3 s without breaking the vacuum conditions. The WCA of the sample pre-treated with H_2_ plasma was significantly low, indicating a super-hydrophilic surface finish (Figure 2a). Additionally, the overall treatment duration reduced from 100 s for O_2_ treatment to only 3 s of O_2_ treatment after H_2_ pre-treatment. Moreover, this method showed the lowest contact angle obtained for PVC compared with previously reported values [63].

**Figure 3:**
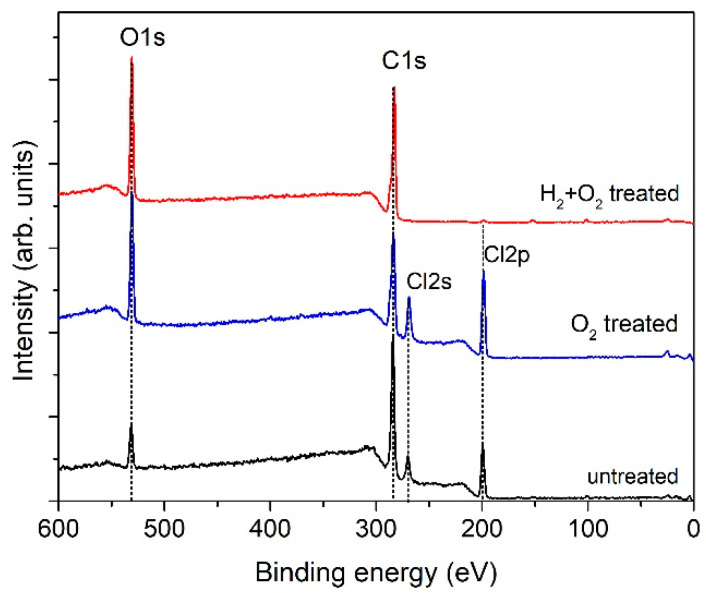
XPS survey spectra of the three samples shown in Figure 2b.

The samples were analysed using XPS to explain the differences in wettability after plasma treatment with and without H_2_ plasma pre-treatment. The XPS composition of the surface film (i.e., the ratio of the elements O/C and Cl/C) is shown in Figure 2b along with images of water droplets on the untreated sample, the sample treated with oxygen plasma afterglow only, and the sample with hydrogen plasma pre-treatment in combination with oxygen plasma afterglow. An example of the corresponding survey spectra is shown in Figure 3. The detailed XPS elemental compositions of the samples deduced using the survey spectra are listed in Table 1. Significant differences were observed after pre-treatment with H_2_ plasma such as a super-hydrophilic surface finish, which is a consequence of the doubly increased oxygen concentration and chlorine depletion (Table 1, Figure 2b and Figure 3). Notably, chlorine concentration was significantly low in the H_2_-pre-treated sample. In contrast, chlorine concentration remained high after treatment with only oxygen plasma afterglow without H_2_ plasma pre-treatment.

**Figure 4:**
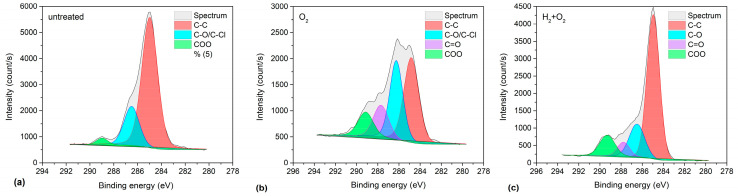
XPS high-resolution (HRES) C1s spectra of: (**a**) as-received PVC foil, (**b**) foil treated in O_2_ plasma afterglow for 100 s, and (**c**) foil pre-treated in H_2_ plasma for 1 s and treated in O_2_ plasma afterglow for 3 s.

XPS high-resolution (HRES) spectra were obtained to evaluate any differences in the types of surface functional groups on the samples with and without hydrogen plasma pre-treatment (Figure 4). Figure 4 shows the carbon C1s spectra corresponding to the sample composition presented in Table 1. Untreated PVC exhibited three peaks at binding energies of 285, 286.5, and 289.3 eV (Figure 4a). The first peak (285 eV) was assigned to C–C/C–H bonds, whereas the second peak (286.5 eV) was assigned to either C–Cl or C–O bonds because the chemical shifts were similar and could not be distinguished from the high-resolution C1s spectra [64]. The last peak (289.3 eV) was assigned to the –COO(H) functional groups. Significant differences were noticed in comparing H_2_-plasma pre-treated samples with non-pre-treated ones. The fitted spectra enabled the evaluation of the concentration of functional groups on the surface film probed using XPS. The results are summarised in Table 2.

Pre-treatment with hydrogen plasma increases the complexity of the surface reactions, which affects the surface finish in terms of both wettability and composition. The most significant effect observed was chlorine depletion. Hydrogen plasma treatment resulted in the formation of a very thin surface film with a composition typical of polyolefins. The photon energy of VUV radiation from hydrogen plasma is in the range of approximately 6–10 eV [60], which was significantly higher than the binding energy between C and Cl in the PVC polymer, which is approximately 3.5 eV [65]. VUV photons break the C–Cl bonds in the surface film, and the dangling bonds are occupied by hydrogen atoms from the plasma because inductively coupled hydrogen plasma is a rich source of H atoms [66]. Thus, the net effect of PVC treatment with powerful hydrogen plasma is the substitution of chlorine with hydrogen in an extremely thin surface film. Additionally, hydrogen plasma causes slow etching of the polymer, which breaks the C–C bonds in the polymer chain; however, the effect of etching is much less intensive than that of chlorine substitution with hydrogen.

Polyolefins are functionalized with polar oxygen-containing functional groups via treatment with oxygen plasma [67]. The dose of oxygen atoms required for maximal wettability varies between different polymer types in the range of 10^23^–10^24^ m^−2^ [68]. In the system used for these experiments, this dose was achieved within a 1 s treatment period with the oxygen plasma afterglow. Furthermore, the rapid functionalization of the samples pre-treated with hydrogen plasma causes significant hydrophilisation, which produces a super-hydrophilic surface finish, as shown in Figure 2.

Notably, the quantity of highly oxidized functional groups (Table 2) was similar between the pre-treated samples and those treated with the oxygen plasma afterglow only. Although pre-treatment with hydrogen plasma produced a super-hydrophilic surface finish (Figure 2), the concentration of highly polar functional groups assigned as COO(H) in Table 2 was similar to that for the samples treated in oxygen plasma afterglow only. The discrepancy between the WCA and XPS results may be attributed to the presence of nonpolar groups associated with chlorine, which were still present in the oxygen-plasma-treated samples. Hence, understanding these effects is important for evaluating very thin surface films that are grafted onto polymer substrates such as chitosan-coated PVC.

Therefore, additional in-depth analyses of surface wettability and surface roughness were performed. It is known that the static contact angle is not a unique value and that the advancing ($\theta_{A}$) and receding ($\theta_{R}$) contact angles associated with liquid spreading must be measured [69]. The advancing contact angle refers to the resistance of the surface to the advancement of a liquid, while the receding angle refers to the resistance of the surface to the retraction of a liquid. A smaller receding angle, therefore, means that the surface attracts the liquid more strongly and prevents it from retracting, which is associated with better adhesion. By measuring the advancing and receding contact angles, the contact angle hysteresis ($\theta_{A}-\theta_{R}$) can be determined, which is a sign of the inhomogeneity of the surface [70]. The results are shown in Table 3. Since hydrophilic surfaces usually have a lower hysteresis, which can lead to the feeling of a more homogeneous surface, we have also added normalized relative values (($\theta_{A}-\theta_{R}$)/$\theta_{A}$) in Table 3 for a more objective comparison of surfaces with large differences in wettability.

**Figure 5:**
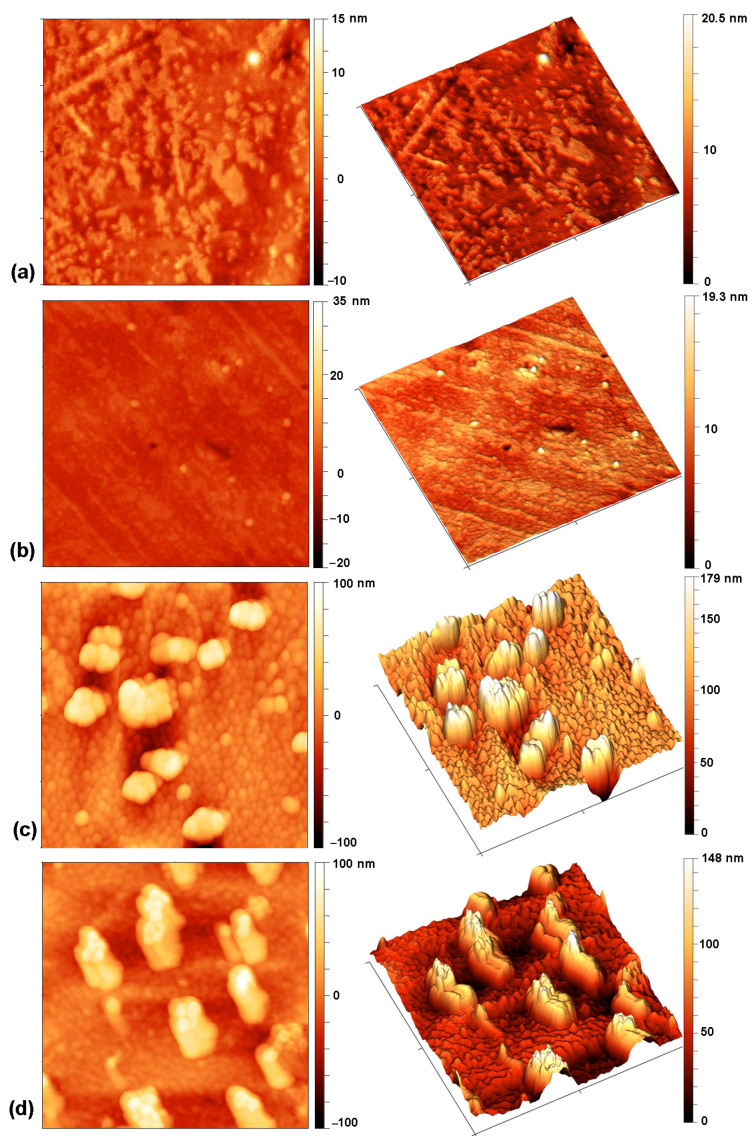
AFM images on an area of 2 µm × 2 µm of: (**a**) as-received PVC foil, (**b**) foil pre-treated in H_2_ plasma for 1 s and treated in O_2_ plasma afterglow for 3 s, (**c**) foil treated in O_2_ plasma afterglow for 100 s before washing, and (**d**) after washing. The roughness parameter S_a_ is 1.5 ± 0.15, 1.2 ± 0.05, 13.9 ± 2.1, and 15.4 ± 5.9 for images presented in (**a**), (**b**), (**c**), and (**d**), respectively.

A comparison of the absolute values of the contact angle hysteresis shows that the untreated sample and the sample treated only with oxygen plasma afterglow (100 s) have a similar hysteresis, while the hysteresis of a sample pre-treated with hydrogen and then treated with oxygen is very low. However, when comparing the relative values, both plasma-treated samples show a higher hysteresis than the untreated samples, which can be attributed to better surface adhesion. If the plasma-treated samples are compared, the sample pre-treated with hydrogen again shows a lower hysteresis. It should be mentioned that adhesion and hysteresis are not directly related but depend on various factors such as homogeneity, roughness, and chemical composition of the surface [71,72]. Figure 5a–c shows typical AFM images of the samples. The images were acquired on several spots in order to evaluate the lateral variations of the roughness. The untreated sample (Figure 4a) and the sample pre-treated in hydrogen plasma with subsequent oxygen treatment for a short time—3 s (Figure 4b)—have quite smooth surfaces with the arithmetical mean height roughness parameter (S_a_) of roughly 1 nm. In contrast, spherical features with a size of 200–400 nm can be observed in the sample treated for 100 s in the oxygen afterglow (Figure 4c). According to the literature, such features may be related to the presence of low molecular weight oxidized material (LMWOM), which can be removed by washing [73,74,75,76]. However, after washing the sample treated for 100 s, these spherical features can still be observed (Figure 5d). Therefore, they cannot be attributed to the formation of LMWOM, but rather to uneven etching. Nevertheless, it is obvious that the surface is inhomogeneous, which is also the reason for the observation of higher hysteresis compared to the sample pre-treated with hydrogen plasma. The combination of surface roughness and moderate hydrophobicity of the sample treated for 100 s makes it difficult to move the contact line of the liquid. The hydrophilic sample pre-treated with hydrogen, which has a low hysteresis, can thus be explained by a more homogeneous and smoother surface (Figure 5b) that allows a liquid to spread more easily.

### 2.2. Deposition of Chitosan on Plasma-Treated PVC Substrates

Based on the hydrophilicity of the plasma-treated polymer samples (Figure 2), we chose the following plasma-treatment conditions for chitosan incubation: (i) treatment time in oxygen plasma afterglow 100 s (minimum WCA was 40°), and (ii) pre-treatment time in hydrogen plasma 1 s then treatment in oxygen plasma afterglow 3 s (WCA was negligibly low).

**Figure 6:**
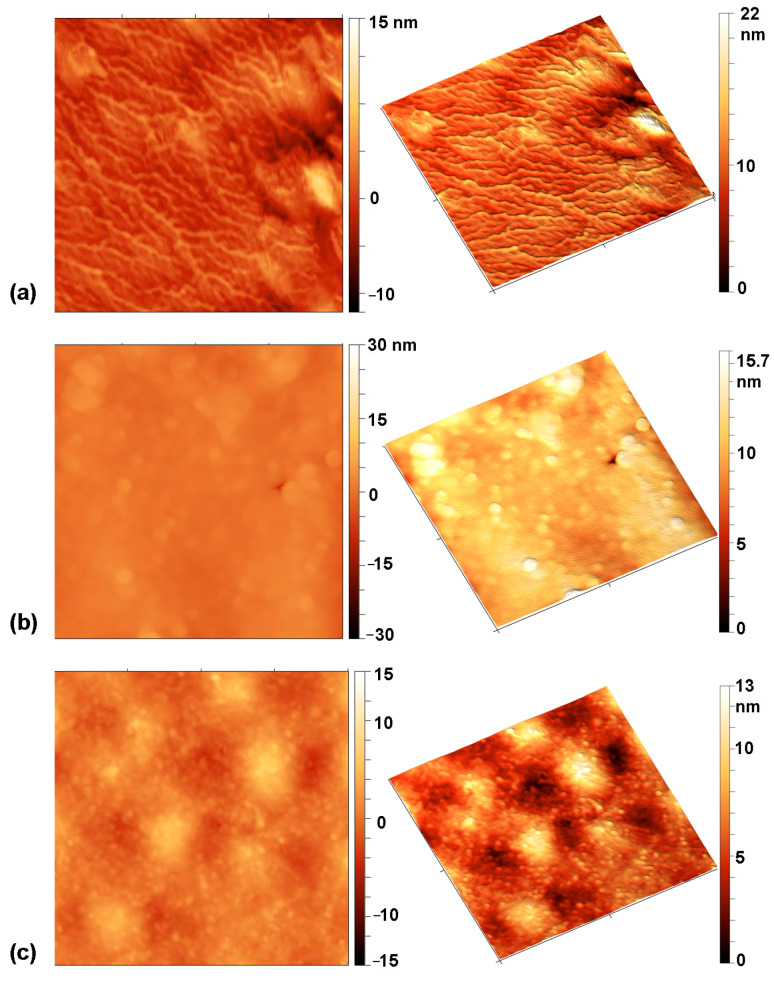
AFM images of chitosan-coated samples: (**a**) as-received PVC foil, (**b**) foil pre-treated in H_2_ plasma for 1 s and treated in O_2_ plasma afterglow for 3 s, (**c**) foil treated in O_2_ plasma afterglow for 100 s. The roughness parameter S_a_ is 1.7 ± 0.14, 1.05 ± 0.1, and 1.47 ± 0.9 for images presented in (**a**), (**b**), and (**c**), respectively.

Chitosan was deposited onto the PVC substrates and activated using both plasma treatment procedures. The AFM images of the chitosan-coated samples are shown in Figure 6. The chitosan is very unevenly distributed on the untreated sample (Figure 6a). On the sample that was treated in oxygen plasma afterglow for 100 s (Figure 6b), the coating is more uniform, and spherical features observed in Figure 5c are almost completely covered with chitosan. In contrast, the chitosan coating on the sample pre-treated with hydrogen and treated in oxygen afterglow for 3 s is very smooth and uniform. This is due to the super-hydrophilic surface, which allows the chitosan solution to spread more easily.

XPS was further used to evaluate the presence of chitosan on the surfaces of the PVC samples. As chitosan contains amino groups, the quantification of nitrogen concentration acts as a good indicator of the presence of chitosan because the other materials used in this study were nitrogen free. The composition of the as-received chitosan powder used in this investigation was 59, 33, and 8 at.% of carbon, oxygen, and nitrogen, respectively. Therefore, monitoring the nitrogen concentration should indicate if a thin chitosan film adhered successfully to the PVC substrates.

**Figure 7:**
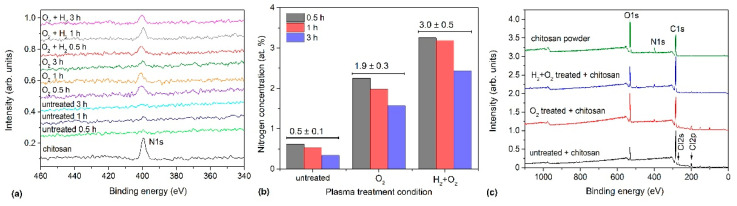
(**a**) Nitrogen peak in XPS spectra for different treatments and various incubation times of 0.5, 1, and 3 h; (**b**) nitrogen concentration for selected treatment conditions; (**c**) example of survey spectra of chitosan-coated samples incubated for 1 h.

Figure 7a shows a segment of the XPS survey spectra measured between 350 and 450 eV binding energies for all samples. Nitrogen appeared at approximately 400 eV binding energy. The chitosan powder spectrum has been included for comparison. The intensity of the N peak is marginal compared with those of other elements, but the results shown in Figure 7a clearly indicate that the nitrogen concentration detected through XPS in the untreated samples on the surface films was at the detection limit of 0.5 at.%. However, the nitrogen concentration in the plasma-treated samples was approximately 2 at.% without hydrogen plasma pre-treatment. Furthermore, nitrogen concentration was still higher at approximately 3 at.% in the H_2_ pre-treated samples activated with 3 s oxygen plasma afterglow. The nitrogen concentrations at different incubation times and treatment conditions are shown in Figure 7b. Incubation time seemed to exert only a minor effect; hence, it has not been discussed here. Although shorter incubation times appeared to be more favourable, the differences were smaller than the detection limit of XPS; therefore, incubation time has been deemed irrelevant to the outcome. However, from an applicability perspective, a shorter incubation time is more convenient.

**Figure 8:**
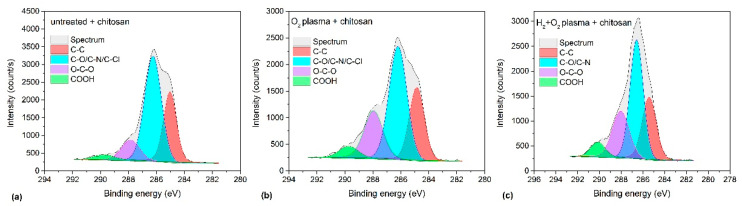
HRES C1s spectra of chitosan-coated samples incubated for 1 h: (**a**) as-received PVC foil, (**b**) foil treated in O_2_ plasma afterglow for 100 s, and (**c**) foil pre-treated in H_2_ plasma for 1 s and treated in O_2_ plasma afterglow for 3 s.

Successful incubation of chitosan on the plasma-treated samples was further confirmed using the obtained HRES C1s spectra shown in Figure 8; the corresponding survey spectra are shown in Figure 7c. A characteristic feature of chitosan is the presence of C–OH and C–NH_2_ groups. Therefore, a peak corresponding to these groups must be dominant in the HRES C1s spectra. Figure 8 distinctly demonstrates this. The peak at 286.2 eV corresponds to C–OH, and C–NH_2_ is the most dominant peak in the spectrum. This peak is accompanied by three other peaks at 258 eV corresponding to C–C/C–H, 287.9 eV corresponding to O–C–O, and 289.6 eV corresponding to COO(H) groups. Notably, although the peak at 286.2 eV appears prominently in the untreated sample, which does not contain chitosan, its appearance may be attributed to the overlapping of the C–Cl component from the substrate but not the presence of chitosan because this sample contains the highest quantity of Cl (Table 4).

Thus, from the spectra shown in Figure 7 and Figure 8, the following conclusions may be drawn: (1) oxygen plasma afterglow treatment improves chitosan immobilisation, (2) additional pre-treatment with H_2_ plasma further enhances chitosan immobilisation, and (3) long incubation times are not required because the intensity of the nitrogen peak does not depend much on the incubation time in the chitosan solution. Considering the nitrogen concentration in the bulk chitosan powder (approximately 8 at.%) and escape depth of photoelectrons in polysaccharides (several nm), we concluded that plasma treatment enabled an almost complete coverage with a thin layer of chitosan as also shown by AFM (Figure 6). Based on the composition of the reference chitosan powder, the nitrogen concentration of 8 at.% would be detected on chitosan-coated samples through XPS if the thickness of the chitosan film were well above the escape depth of photoelectrons (>10 nm). In contrast, the detected nitrogen concentration would be marginal if a significant area was not covered with chitosan because the XPS signal would be averaged over the covered and uncovered areas. Thus, the signal from the monolayer should lie between these two extremes. Different models have been proposed for estimating the thickness of a monolayer [77,78,79,80], and the results vary; however, generally, a monolayer should exhibit 2–3 times lower nitrogen concentration than that of a thick film. As we detected 3 at.% nitrogen (2–3 times lower than the N concentration in bulk chitosan), we have assumed an almost complete coverage of the polymer substrate with the chitosan monolayer in the case of pre-treatment with hydrogen plasma followed by treatment in oxygen-plasma afterglow.

Chitosan is renowned for its ability to form hydrogen-bonding and electrostatic interactions. Its mechanisms of adhesion were explained in our previous papers [81,82]. The interactions are maximized by an attractive force between a hydrogen atom bonded to an electronegative atom on the surface of the substrate. The plasma treatment causes the super-hydrophilic surface finish, which provides highly polar and charged surface functional groups (including OH and COOH groups), and these groups are likely to provide interaction sites for hydrogen bonds with chitosan. Alternatively, Renoud et al. [83] showed the possibility of forming a covalent bond between the amino group in chitosan and a carbonyl group (which can be formed also on plasma-treated samples) on the surface of triethoxysylilbutyraldehyde. The authors studied the strength of the bond between chitosan and the carbonyl group from polymer and found excellent scratch resistance. No delamination was observed, and the authors reported only progressive plastic deportation during the scratch tests. The stability of chitosan bonded with electrostatic ionic interactions was also investigated for chitosan-coated polyester fabric after washing cycles [84]. For untreated fabric, the chitosan coating was removed after 10 washing cycles. For fabric modified by hydrolyzes and ionic promoters that increased ionic interactions with chitosan, the coating remained stable after washing. Here it should be mentioned that urinary catheters are intended only for a single use of a short duration (order of minutes) just to empty the bladder. Therefore, we can assume that the chitosan coating is stable enough for this application.

### 2.3. Adhesion and Proliferation of Urothelial Cells on Chitosan-Coated PVC Substrates

Figure 7 and Figure 8 and Table 3 indicate significant modification of the PVC substrates after treating the foils with hydrogen plasma and oxygen plasma afterglows and incubation in chitosan solution. The differences in nitrogen concentration (which reflect the chitosan quantity on the surface) for different plasma treatments (Figure 7) might affect urothelial cell adhesion [85,86,87,88,89], so we performed biological tests by imaging the samples incubated with the urothelial cells and evaluation of the metabolic activity of those cells.

#### 2.3.1. Metabolic Activity

**Figure 9:**
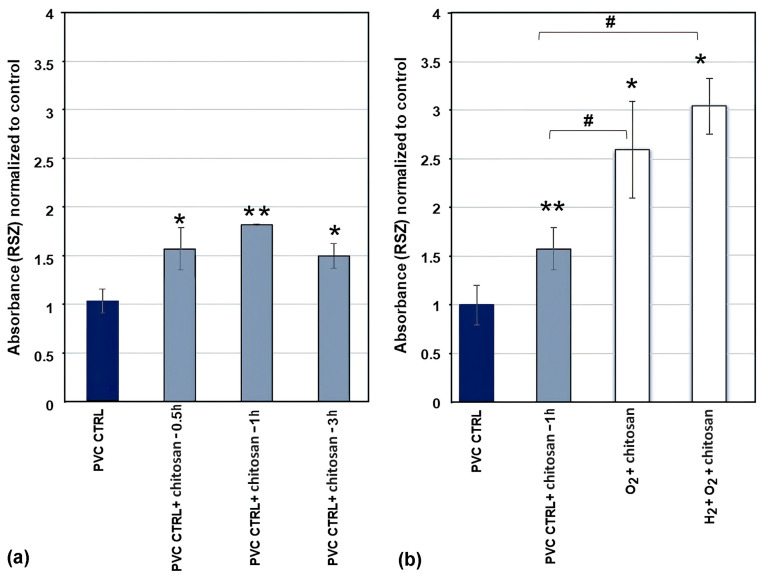
Metabolic activity of adherent urothelial cells on various plasma-treated samples incubated in chitosan solution—measured using resazurin absorbance assay 4 h after seeding the cells. (**a**) Untreated samples (PVC CTRL) immersed into chitosan solution for 0.5, 1, and 3 h (samples denoted as PVC CTRL + chitosan-0.5h, PVC CTRL + chitosan-1h, and PVC CTRL + chitosan-3, respectively); (**b**) plasma-treated samples immersed into the chitosan solution for 1 h (samples denoted as O_2_ + chitosan and H_2_ + O_2_ + chitosan). Two-way ANOVA was used, *^, #^ *p* < 0.05, ** *p* < 0.01 (*, ** compared to control sample PVC CTRL, # compared to PVC CTRL + chitosan).

The adhesion of biological cells was estimated by measuring the metabolic activity of the adherent urothelial cells using a resazurin assay, and the results are shown in Figure 9. Figure 9a shows the metabolic activity of adherent urothelial cells on uncoated PVC substrates (denoted as PVC CRTL) and untreated PVC substrates immersed in the chitosan solution for different time durations (denoted as PVC CRTL + chitosan). Notably, immersion in untreated PVC substrates caused a statistically significant increase in metabolic activity. This was not consistent with the results shown in Figure 7, which showed a marginal (if any) concentration of nitrogen on the surface of the untreated PVC substrate. This paradox may be attributed to the sensitivity of XPS to nitrogen. Some chitosan may have been adsorbed onto the untreated substrates; however, its concentration was below the XPS detection limit. Additionally, Figure 9a shows that the metabolic activity of cells in chitosan-incubated untreated samples was not affected by the incubation time period in the chitosan solution. Nevertheless, the highest increase in adhesion rate was observed for 1 h chitosan treatment (Figure 9a); hence, this incubation time was used in further experiments involving plasma-treated samples.

The metabolic activity of the biological cells on chitosan-coated plasma-treated PVC substrates has been summarised in Figure 9b. These results distinctly show that chitosan and plasma treatments exert significant effects on cell adhesion onto PVC substrates. The metabolic activity of cells seeded onto the plasma-treated samples was approximately twice that of the untreated samples. Moreover, the metabolic activity of urinary cells was high for the samples pre-treated with hydrogen plasma; however, the error bar shown in Figure 9b was large; therefore, the difference is not conclusive. From this perspective, we concluded that the level of hydrophilisation does not govern the metabolic activity of the urinary cells. A huge difference was observed in the WCA between samples treated with oxygen plasma afterglow only (WCA, approximately 40°) and those subjected to additional pre-treatment with hydrogen plasma (negligibly low WCA) (Figure 2), whereas the difference in metabolic activity (Figure 9b) was minor (within the statistical error).

#### 2.3.2. Visualisation and Confluence

**Figure 10:**
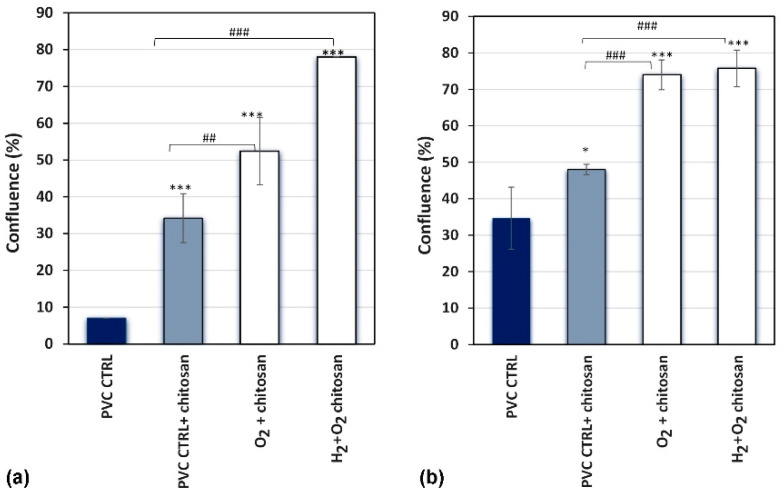
Cell confluency measurements of plasma-treated samples immersed into chitosan solution (samples O_2_ + chitosan and H_2_ + O_2_ + chitosan) at (**a**) 4 h and (**b**) 24 h after seeding urothelial cell; measurement was performed using EVOS M5000 Cell Imaging System (Thermo Fisher Scientific, Waltham, MA, USA). Two-way ANOVA was used, * *p* < 0.05; ^##^ *p* < 0.01, and ***^, ###^ *p* < 0.001 (* compared to PVC control, # compared to PVC control + chitosan).

The cells were visually checked for adherence to chitosan- and plasma-treated samples. Thus, cell confluence was assessed 4 and 24 h after cell seeding on samples treated with different plasma solutions and incubated in chitosan for 1 h. Before imaging the cells using an inverted microscope, the samples were thoroughly washed with PBS to remove all nonadherent cells. A fresh cell culture medium was added to the samples. Confluence (percentage of surface area covered by adherent cells) was evaluated in four independent wells for each sample. The results of the confluence assessment are shown in Figure 10 as the average measurements for all four wells.

**Figure 11:**
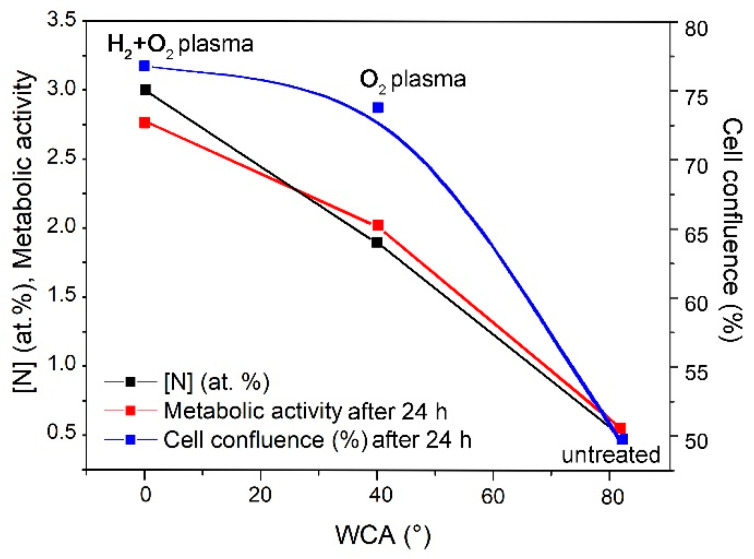
Intercorrelation of nitrogen concentration, cell confluence, and metabolic activity after 24 h versus wettability.

Increased cell confluence was observed in all chitosan- and plasma-treated samples compared with the control at 4 h after cell seeding (Figure 10a). The control samples (without immersion in chitosan solution and without plasma treatment) exhibited the lowest confluence of approximately 7%. Immersion of untreated samples in chitosan solution was beneficial because approximately 35% of the sample area was covered with the cells. This implies that even a small quantity of chitosan (XPS showed a negligibly low nitrogen concentration; Figure 7) enabled several times better adhesion of the cells at 4 h after seeding. A comparison of the results after plasma treatments with that after chitosan immersion alone showed that the highest cell confluence of approximately 75% was observed in samples treated with a combination of H_2_ and O_2_ plasmas, whereas treatment with O_2_ plasma afterglow alone showed a 50% confluence rate, which demonstrates the additive effect of H_2_ plasma pre-treatment on chitosan deposition and cell adhesion. These results (shown in Figure 10a) qualitatively concur with the results of the resazurin assay, which reflect the metabolic activity of adhered cells (Figure 9b), nitrogen concentration (Figure 7), and surface wettability (Figure 2). This correlation is illustrated in Figure 11.

The differences in cell confluence were not as significant for up to 24 h after seeding the urothelial cells (Figure 10b). The confluence of cells on the chitosan-coated samples was higher than that on the as-received PVC samples; however, the difference was not significant between the samples pre-treated with hydrogen plasma and those treated with oxygen plasma afterglow only. This result was expected because the cells eventually covered the entire surface.

**Figure 12:**
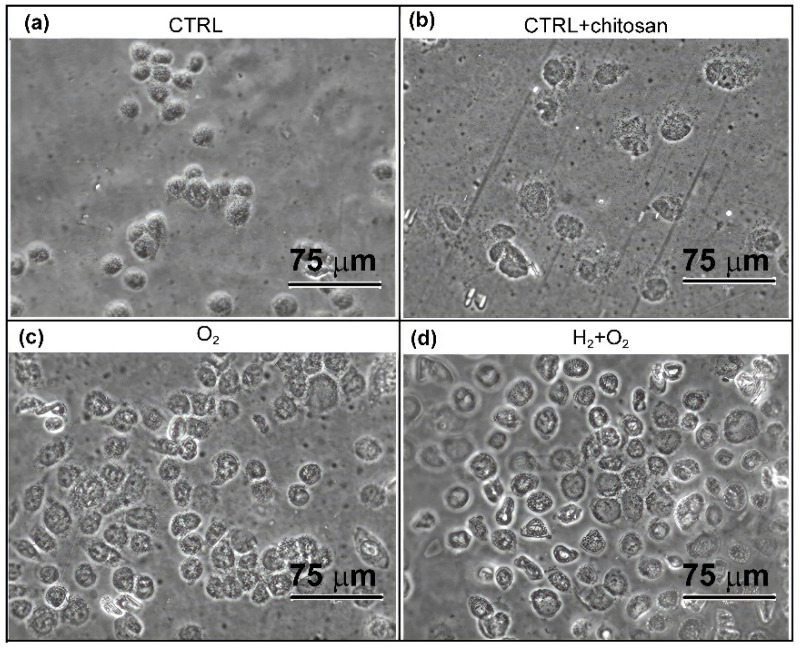
Representative images of RT4 cell growth 4 h after seeding. (**a**) As-received PVC substrates, (**b**) substrates immersed in chitosan solution, (**c**) treated with oxygen plasma afterglow and immersed in chitosan solution, (**d**) pre-treated with hydrogen plasma, treated with oxygen plasma afterglow, immersed in chitosan solution. Images were captured using an EVOS 5000 light microscope (Thermo Fisher Scientific, Waltham, MA, USA) at 400× magnification. The scale bar is 75 µm.

Cell morphology was monitored for all the samples, and the results are shown in Figure 12. Cells adhering to the as-received PVC samples (without chitosan coating and plasma treatment, Figure 12a) exhibited a highly round morphology after 4 h of seeding with little tendency to spread. Chitosan immobilisation on the PVC surface elicited a pronounced improvement in cell spreading in all samples. As observed distinctly in Figure 12b–d, the cells spread on the surface of the samples immersed in the chitosan solution because of their active morphology.

**Figure 13:**
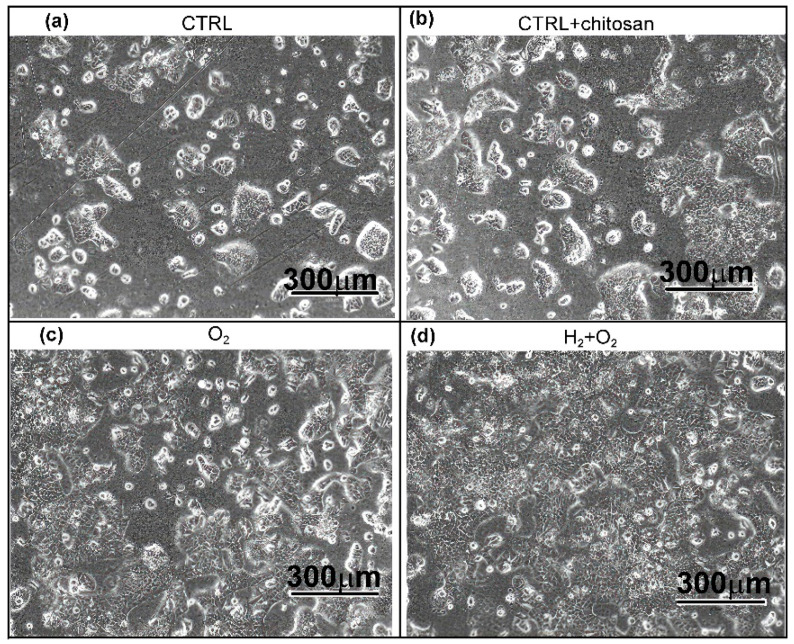
Representative images of RT4 cell growth 24 h after seeding. (**a**) As-received PVC substrates, (**b**) substrates immersed in chitosan solution, (**c**) treated with oxygen plasma afterglow and immersed in chitosan solution, (**d**) pre-treated with hydrogen plasma, treated with oxygen plasma afterglow, and immersed in chitosan solution. Images were captured using EVOS 5000 light microscope (Thermo Fisher Scientific, Waltham, MA, USA) at 100× magnification. The scale bar is 300 µm.

Figure 13 shows images of the samples at 24 h after seeding. The urothelial RT4 cells showed growth in clusters as is the norm for the cell type. Their shapes were compact, and they adhered closely to each other, which is typical of epithelial cells. Overall, Figure 12 and Figure 13 clearly show that chitosan coating promotes the attachment and growth of RT4 cells on the surface, whereas plasma treatments refined the surface for optimal chitosan coating.

The plasma treatment for tailoring surface properties of polymers has certain limitations. As shown in this paper, the treatment with oxygen atoms from plasma (a standard procedure for functionalization of polymers with highly polar functional groups) does not enable the depletion of chlorine, so we had to use VUV radiation arising from hydrogen plasma for breaking the C-Cl bonds and thus ensuring the appropriate surface finish. The treatment conditions should be optimized. As shown in this article, the prolonged treatment with oxygen atoms causes modifications of the surface morphology, which may be an obstacle to the functional properties of catheters. Furthermore, hydrogen plasma treatment for a very short time will not ensure the appropriate removal of chlorine atoms from the surface layer of PVC, while longer treatment times will cause significant heating and, thus, thermal degradation of PVC.

Thus, immobilisation of chitosan on plasma-treated samples represents an ecologically benign method for improving the biocompatibility of PVC-based urinary catheters. Applicability in medical practice is only possible through extensive clinical tests, which are beyond the scope of this study, as are the technological challenges in upscaling these methods. The selected discharge power density for sustaining oxygen and hydrogen plasma is probably too large for mass application; therefore, energy efficiency needs to be optimized.

## 3. Materials and Methods

### 3.1. Plasma Treatment Procedure

Unplasticized polyvinyl chloride (PVC) polymer foil (thickness: 0.2 mm) was purchased from Goodfellow Ltd. (Huntingdon, UK). The PVC foil was cut into 1 cm diameter disks and treated with low-pressure plasma. The experimental plasma system used for surface activation of polymer foils has been discussed in our previous paper [58]. The plasma system consisted of an 80 cm long borosilicate glass discharge tube pumped with a two-stage rotary pump at a nominal pumping speed of 80 m^3^/h. The discharge tube was 4 cm in diameter, and a coil with 6 turns was used to sustain the plasma. The coil was connected to a radiofrequency (RF) generator (13.56 MHz) through a matching network, and forward power was set to 500 W. The samples were placed in the afterglow region, which was 30 cm away from the coil downstream of the gas flow, and treated with O_2_ plasma flowing afterglow. O_2_ pressure was set to 30 Pa, and the flow rate was 105 sccm. The treatment time in the oxygen plasma afterglow ranged from 1 to 200 s. The samples treated in the afterglow remained at room temperature even after prolonged treatment. Another set of samples was pre-treated for 1 s with VUV radiation from H_2_ plasma. The discharge power for the hydrogen plasma was set to 400 W, pressure to 22 Pa, and hydrogen flow rate to 140 sccm. During pre-treatment with glowing hydrogen plasma, the samples were placed inside the coil. After pre-treating, they were treated in oxygen plasma afterglow for treatment times ranging from 1 to 200 s.

### 3.2. Surface Wettability

The wettability of the plasma-treated PVC foils was measured with respect to treatment time to determine the optimal treatment conditions for achieving the best hydrophilicity. A drop shape analyser (DSA 100, Krüss GmbH, Hamburg, Germany) was used to perform water contact angle (WCA) measurements. The static contact angle was measured using the sessile drop method [90]. The volume of a drop was set to 2 µL. Milli-Q water was used to determine wettability. Several measurements (three to five) were performed for each sample. The samples showing optimal wettability were selected and used for chitosan deposition and cell incubation (described in Section 2.3). For these selected samples, the advancing and receding contact angles were additionally measured to determine the contact angle hysteresis.

### 3.3. Deposition of Chitosan

Chitosan powder was purchased from Sigma–Aldrich (Darmstadt, Germany). A 1.5% (*w*/*w*) chitosan solution was prepared using Milli-Q water, by following a previously described procedure [91]. Chitosan is water-soluble depending on its molecular weight and acetylation degree as well as on the pH of the media. We used low molecular weight chitosan (50–190 kDa) with deacetylation degree ≥ 75%. Because chitosan samples with higher molecular weights are only soluble in acidic aqueous media, we adjusted the pH to 4 by adding HCl. A 100 µL drop of chitosan solution was placed on the sample surface and transferred to an incubator shaker (Eppendorf New Brunswick Innova 42, Eppendorf, Hamburg, Germany). The sample was processed at 27 °C for different time durations (30 min, 1 h, and 3 h) for even distribution of chitosan on the surface, followed by drying. Subsequently, the samples were rinsed thoroughly with Milli-Q water to remove excess (unbonded) chitosan. Then, the samples were placed inside an incubator shaker for 30 min to dry, followed by rinsing. This step was repeated until the samples were rinsed thrice.

### 3.4. Surface Morphology

The surface morphology of the plasma-treated samples was measured using atomic force microscopy (AFM) with an NT-MDT instrument (Moscow, Russia). The measurements were performed in a semi-contact mode using the NT-MDT silicon tips and a recording frequency of 1 Hz. The AFM images were acquired over an area of 2 × 2 and 20 × 20 µm^2^. The surface roughness was calculated and expressed as average roughness (Ra).

### 3.5. Chemical Composition

The plasma-treated samples before and after chitosan deposition were characterised using X-ray photoelectron spectroscopy (XPS) with a TFA XPS (Physical Electronics, Munich, Germany) instrument. XPS is a surface-sensitive method with a detection depth of a few nanometres; hence, it is used as the standard method for characterising surfaces and thin films [92]. The samples were exposed to monochromatic Al Kα_1,2_ radiation at a photon energy of 1486.6 eV. The spectra were recorded at an electron take-off angle of 45°. The survey spectra were acquired at a pass energy of 187 eV using an energy step of 0.4 eV. High-resolution C1s spectra were measured at a pass energy of 29.35 eV using an energy step of 0.125 eV. An additional electron gun was used to compensate for the accumulation of surface charge. The C1s peak corresponding to C–C bonds was set at 285 eV. The measured spectra were analysed using the MultiPak v8.1c software provided with the spectrometer.

### 3.6. Resazurin Reduction-Based Cell Viability Assay

Resazurin reduction-based assays were performed to explore the effects of chitosan coating on the viability of RT4 cells (human urinary bladder cell line). Resazurin is a widely used biochemical assay in cell biology for measuring cell viability through its conversion into a fluorescent dye. The measured fluorescence intensity provides an estimate of the number of viable cells [93]. The RT4 cells were seeded onto 24-well plates in 500 μL of culture media (1 × 10^5^ cells/mL). A Dulbecco’s modified Eagle’s medium (A-DMEM)/F12 (1:1) containing 5% heat-inactivated fetal bovine serum (FBS), 10,000 U/mL penicillin, and 10,000 µg/mL streptomycin solution was used as the culture medium. The RT4 cells were incubated for 4 and 24 h on the PVC samples. After incubation, the cell medium was removed, and the samples were rinsed and transferred to another 24-well plate. Fresh culture medium was added to each well with a sample (500 µL/well). Metabolic activity and cell proliferation were assessed by performing the resazurin assay. Briefly, 100 μL of 0.4 mg/mL of resazurin solution (Sigma-Aldrich, Darmstadt, Germany). was added to each well and incubated for 5 h prior to measurement of absorbance (Tecan, Männedorf, Switzerland) at 578 nm and 630 nm. Untreated PVC samples with no chitosan immobilised on the surface were used as background controls. The metabolic activity of urothelial cells was normalised to the absorbance of the controls.

The morphology of the adhered urothelial cells was investigated using an EVOS 5000 inverted microscope (Thermo Fisher Scientific, Waltham, MA, USA). Before imaging, the samples were thoroughly washed in phosphate-buffered saline (PBS) to remove all non-adherent cells and transferred to a fresh cell medium. Percent (%) confluence was evaluated in four independent wells using the EVOS 5000 Cell Imaging System software (version Rev. 1.7.2401.174).

### 3.7. Statistical Analysis

Data on cell metabolic activity and confluency were statistically analysed using JASP 0.16.3.0. open-source software (University of Amsterdam, Amsterdam, The Netherlands). Group means were calculated and compared using an ANOVA followed by a post-hoc Tukey’s range test. Differences in means were considered statistically significant at *p* < 0.05, *p* < 0.01, and *p* < 0.001. Error bars in the charts represent standard errors.

## 4. Conclusions

Chitosan coatings on medical devices such as urinary catheters improve urothelial cell adhesion and promote cell growth. In this study, medical-grade PVC foils were used to study the adhesion of the chitosan film and the associated biological responses. The PVC foils were treated with gaseous plasma to enhance chitosan immobilisation. Plasma in the H-mode was sustained using an electrodeless RF discharge. Two types of plasma treatment were used: (1) classical treatment using oxygen plasma early flowing afterglow for 100 s and (2) innovative pre-treatment using a dense hydrogen plasma sustained by an electrodeless radiofrequency discharge in the H mode for 1 s, followed by brief treatment with oxygen plasma afterglow for 3 s. The first treatment produced a moderately hydrophilic surface finish with a water contact angle of 40°, whereas the second resulted in a super-hydrophilic surface finish (negligibly low water contact angle). The samples were immersed in chitosan solution at 1.5% (*w*/*w*) concentration and pH 4, followed by thorough rinsing with distilled water to remove unbonded chitosan. Chitosan immobilisation was estimated by measuring the nitrogen concentration on the sample surface using XPS. The concentration depended on the plasma treatment conditions and reached up to approximately 4 at.%. The untreated PVC samples immobilised only low quantities of chitosan (the N concentration estimated using XPS analysis was below the detection limit of approximately 0.5 at.%), whereas moderately hydrophilic surfaces immobilised a notable quantity of chitosan (approximately 1–2 at.%). The highest quantity of chitosan corresponded to an XPS nitrogen concentration of 4 at.% and was observed on super-hydrophilic substrates. The biological response was studied by visualisation, confluence estimation, and assessment of the metabolic activity of urothelial cells, which increased by 2–3 times that observed for untreated catheters. Immersion of the substrates in chitosan solution was beneficial for cell proliferation even for untreated PVC foils. The plasma treatments further enhanced the biocompatibility. A good correlation was observed between surface wettability, nitrogen concentration on the surface, metabolic activity, and cellular confluence on the samples. These results indicate that the super-hydrophilic surface finish of PVC provides optimal conditions for chitosan immobilisation, which enhances the biocompatibility of these materials that are commonly used for fabricating urinary catheters.

## Figures and Tables

**Table 1:** Surface elemental composition and water contact angle (WCA) of samples that were untreated, treated in oxygen plasma afterglow for 100 s, and pre-treated in hydrogen plasma for 1 s and oxygen plasma afterglow for 3 s.

Pre-Treatment	Treatment	C (at.%)	O (at.%)	Cl (at.%)	O/C	Cl/C	WCA (°)
none	untreated	78.8	10.2	11.0	0.13	0.14	82.0
none	O_2_ (100 s)	60.9	24.3	14.8	0.40	0.24	38.0
H_2_ (1 s)	O_2_ (3 s)	71.6	28.0	0.4	0.39	0.01	<2

**Table 2:** Functional groups based on fitted high-resolution C1s spectra.

Pre-Treatment	Treatment	C–C (%)	C–O/C–Cl (%)	C=O (%)	COO(H) (%)
none	none	79.5	17.8		2.7
none	O_2_ (100 s)	42.2	36.8	11.0	10.0
H_2_ plasma (1 s)	O_2_ (3 s)	65.0	18.8	6.9	9.3

**Table 3:** Comparison of static ($\theta_{static}$), advancing ($\theta_{A}$), and receding ($\theta_{R}$) contact angles of selected plasma-treated samples, contact angle hysteresis ($\theta_{A}$−$\theta_{R}$), and roughness (R_a_).

Pre-Treatment	Treatment	$\theta_{static}$ (°)	$\theta_{A}$ (°)	$\theta_{R}$ (°)	($\theta_{A}$−$\theta_{R}$) (°)	$\frac{\theta_{A}-\theta_{R}}{\theta_{A}}$	R_a_ (nm)
none	untreated	82.0	89.9	66.6	23.3	0.26	1.5
none	O_2_ (100 s)	38.0	42.3	17.5	24.8	0.59	13.9
H_2_ (1 s)	O_2_ (3 s)	<5	8.4	4.5	3.9	0.46	1.2

**Table 4:** Surface composition of chitosan-coated samples.

Pre-Treatment	Treatment	C (at.%)	N (at.%)	O (at.%)	Cl (at.%)
none	none	80.8	0.6	14.4	4.2
none	O_2_	72.6	2.2	21.4	3.8
H_2_ plasma	O_2_	75.1	3.2	20.8	0.9

## Data Availability

Data available upon request from the authors.

## Abbreviations

The following abbreviations are used in this manuscript:

PVC	Polyvinyl chloride
WCA	Water contact angle
VUV	Vacuum ultraviolet radiation
AFM	Atomic force microscopy
SEM	Scanning electron microscopy
XPS	X-ray photoelectron spectroscopy
HRES	High-resolution XPS spectra
LMWOM	Low-molecular weight-oxidized material
CTRL	Control sample (untreated PVC)
RT4	Human urinary bladder cell line
FBS	Fetal bovine serum
PBS	Phosphate-buffered saline
DMEM	Dulbecco’s Modified Eagle Medium
RF	Radiofrequency
